# Chick Lrrn2, a novel downstream effector of Hoxb1 and Shh, functions in the selective targeting of rhombomere 4 motor neurons

**DOI:** 10.1186/1749-8104-4-27

**Published:** 2009-07-14

**Authors:** Laura C Andreae, Andrew Lumsden, Jonathan D Gilthorpe

**Affiliations:** 1 MRC Centre for Developmental Neurobiology, King's College London, New Hunt's House, Guy's Campus, London, SE1 1UL, UK; 2 Umeå Centre for Molecular Medicine, Umeå University, 901 87 Umeå, Sweden

## Abstract

**Background:**

Capricious is a *Drosophila *adhesion molecule that regulates specific targeting of a subset of motor neurons to their muscle target. We set out to identify whether one of its vertebrate homologues, Lrrn2, might play an analogous role in the chick.

**Results:**

We have shown that *Lrrn2 *is expressed from early development in the prospective rhombomere 4 (r4) of the chick hindbrain. Subsequently, its expression in the hindbrain becomes restricted to a specific group of motor neurons, the branchiomotor neurons of r4, and their pre-muscle target, the second branchial arch (BA2), along with other sites outside the hindbrain. Misexpression of the signalling molecule Sonic hedgehog (Shh) via *in ovo *electroporation results in upregulation of *Lrrn2 *exclusively in r4, while the combined expression of Hoxb1 and Shh is sufficient to induce ectopic *Lrrn2 *in r1/2. Misexpression of Lrrn2 in r2/3 results in axonal rerouting from the r2 exit point to the r4 exit point and BA2, suggesting a direct role in motor axon guidance.

**Conclusion:**

Lrrn2 acts downstream of Hoxb1 and plays a role in the selective targeting of r4 motor neurons to BA2.

## Background

The hindbrain is patterned during development along two major axes, anterior-posterior (AP) and dorsal-ventral (DV). This generates a Cartesian coordinate system in which, theoretically, the fate of an individual cell, or group of cells, can be specified by its position relative to these orthogonal axes of positional information [1]. The key regulators of AP patterning for the posterior central nervous system (CNS) are the *Hox *genes, which are expressed in a segmental fashion in the hindbrain and have anterior expression boundaries that correspond to specific rhombomere boundaries [1-4]. Much as their *Drosophila *homologues confer segment-specific identity upon the body segments of the fly embryo, the vertebrate *Hox *genes confer segment-specific identity upon rhombomeres: gain or loss of function results in homeotic transformations [5-10]. Along the DV axis different cell fates are regulated by secreted signalling molecules. Of these, by far the best-studied system is the patterning of the ventral neural tube by graded concentrations of Sonic hedgehog (Shh) [11-13]. Shh is expressed by the floor plate and notochord, and regulates the expression of a series of homeodomain transcription factors, which in turn generate sharp domains of expression by cross-repressive effects [11,14,15]. Specific and distinct neuronal cell types are then generated from these domains. For example, in the spinal cord, one class of interneuron (V_0_) develops from the region immediately adjacent to the floor plate, while motor neurons are generated from the next most ventral domain.

While the transcriptional components that respond to early patterning signals are increasingly understood, less is known about the downstream targets that directly regulate cellular behaviour, such as specific axonal projections. For example, *Hoxb1 *is expressed at early stages throughout rhombomere 4 (r4), and loss-of-function mutations in the mouse lead to marked abnormalities of the r4-derived motor neurons [7,16]. Furthermore, misexpression of Hoxb1 in basal r2 can result in a transformation of the presumptive r2 motor neurons to an r4-like identity (that is, trigeminal to facial), as assessed by the re-routing of these r2 motor projections from their usual destination of the first branchial arch (BA1) to the facial motor neuron target, BA2 [6]. The misexpression of Hoxb1 must result in critical changes to the cell surface molecules expressed by these motor neurons, such that their axons follow guidance cues appropriate to those of r4. *Hoxb1 *has the capacity to regulate its own expression (autoregulation), the expression of other *Hox *genes (cross-regulation) and a number of other transcriptional control genes, such as *GATA2*, *GATA3 *and *Phox2b *[6,17-19]. Recently, a microarray screen in zebrafish has identified a number of Hoxb1 downstream targets [20]. However, the identities of downstream effector genes, particularly those encoding cell surface guidance molecules, remain largely unknown.

Several generic guidance cues – that is, those acting on all types of motor neuron – have been identified, including chemoattractant cues from their targets [21-24]. However, the existence also of specific cues is made apparent by phenomena such as the misrouting of Hoxb1^+ ^r2 motor neurons [6], or the abnormal trajectories taken by r5 facial branchiomotor axons to reach their original target when grafted ectopically [25]. Many of the ligand-receptor systems involved in axon guidance are evolutionarily conserved between invertebrate and vertebrate species [26,27] and several molecules that regulate specific axon targeting events have been identified in *Drosophila*. For example, the leucine-rich repeat-containing molecules Connectin and Capricious (Caps) are expressed in specific subsets of motor neurons and their muscle targets and regulate axonal targeting in these contexts [28-31]. The closest vertebrate relatives of Caps are members of the Lrrn (leucine-rich repeat neuronal) family [32-35]. In this study, we show that one member of this family, *Lrrn2*, is expressed in r4 motor neurons and their target, the mesodermal pre-muscle plate of BA2 in chick. Furthermore, we demonstrate that *Lrrn2 *expression is regulated by both Hoxb1 and Shh, and that misexpression of Lrrn2 in r2/3 can result in routing abnormalities of motor axons. Thus, Lrrn2 appears to function in r4 motor neurons downstream of Hoxb1 and Shh to integrate both AP and DV signalling components and to participate in the peripheral targeting of r4 motor axons.

## Results

### *Lrrn2 *is expressed in presumptive r4 territory from early stages

**Figure 1:**
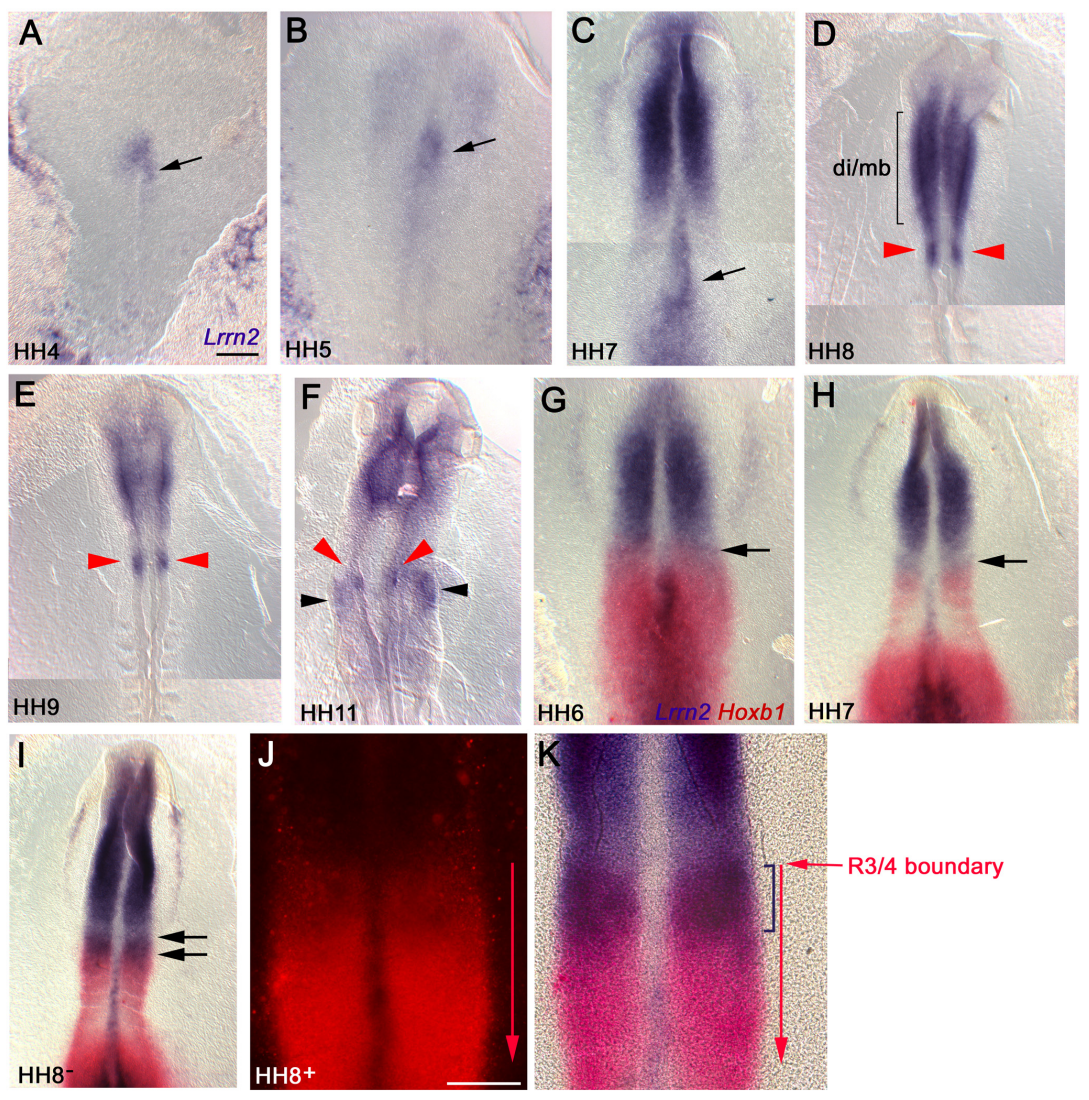
**Time-course of *Lrrn2 *expression**. Chick embryos from HH4–11 following *in situ *hybridisation for *Lrrn2 *and *Hoxb1*. All views are dorsal side up, anterior to the top. **(A) **HH4, **(B) **HH5, **(C) **HH7. Note asymmetric expression of *Lrrn2 *on the right side of the node (arrows in (A-C)). **(D) **HH8, **(E) **HH 9, **(F) **HH11. Expression of *Lrrn2 *starts to localise to the r4 region at HH8 and by HH9 is clearly distinguishable (red arrowheads in (D, E)). Strong staining is also seen in the prospective diencephalon-midbrain region (di/mb) (D). At HH11, r4 expression within the neuroepithelium can be seen (red arrowheads), along with expression in the adjacent mesoderm (black arrowheads in (F)). **(G-K) **Double *in situ *hybridisation with *Lrrn2 *(blue) and *Hoxb1 *(red). (G) HH6, (H) HH7; arrows indicate anterior boundary of *Hoxb1 *expression (future r3/4 boundary). (I) HH8^-^: the region between the double, black arrows highlights overlap in expression, corresponding to presumptive r4. (J) HH8^+^: high power view of hindbrain region with fluorescent image of *Hoxb1 *expression where the r3/4 boundary is clear, despite some quenching due to the NBT/BCIP staining of *Lrrn2*, and (K) brightfield image showing the same. The anterior boundary of the *Lrrn2 *stripe (bracket) coincides with the anterior boundary of *Hoxb1*, indicating that *Lrrn2 *is expressed in early r4. Scale bars: 100 μm.

*Lrrn2 *is first expressed in chick embryos at Hamburger and Hamilton stage 4 (HH4) in a triangular region around Hensen's node (Figure 1A). Expression is activated in the neural plate at HH5 (Figure 1B), becoming more robust by HH7, when expression also extends to posterior regions (Figure 1C). A striking feature during these early stages is its asymmetric expression on the right-hand side of the node (Figure 1A–C). By HH8, the expression pattern has become increasingly restricted: strong staining is visible in the prospective forebrain (diencephalon), midbrain and hindbrain, with a posterior domain of stronger expression becoming delineated (Figure 1D). By HH9, this domain has resolved into a stripe that corresponds to prospective r4 (Figure 1E). By HH11, expression is visible not only in r4, but also in the adjacent mesoderm (Figure 1F). Midbrain and diencephalic expression of *Lrrn2 *persists throughout these stages.

To better define the location of the developing stripe of *Lrrn2 *expression in the hindbrain, double-labelled *in situ *hybridisation was performed using a chick *Hoxb1 *probe (Figure 1G–I). At these early stages, the anterior border of *Hoxb1 *expression corresponds to the r3/r4 boundary [36]. At HH6–7, the posterior border of *Lrrn2 *expression was not distinct but showed a clear overlap with *Hoxb1 *(Figure 1G, H). By HH8, *Lrrn2 *upregulation was evident in a domain coincident with the anterior-most staining of *Hoxb1 *(Figure 1I–K). Thus, *Lrrn2 *is expressed in the early neural plate with a posterior border that approximates to future r4 at early stages and later becomes refined specifically to r4.

### *Lrrn2 *labels post-mitotic motor neurons in r4 and their corresponding target tissue in the second branchial arch

**Figure 2:**
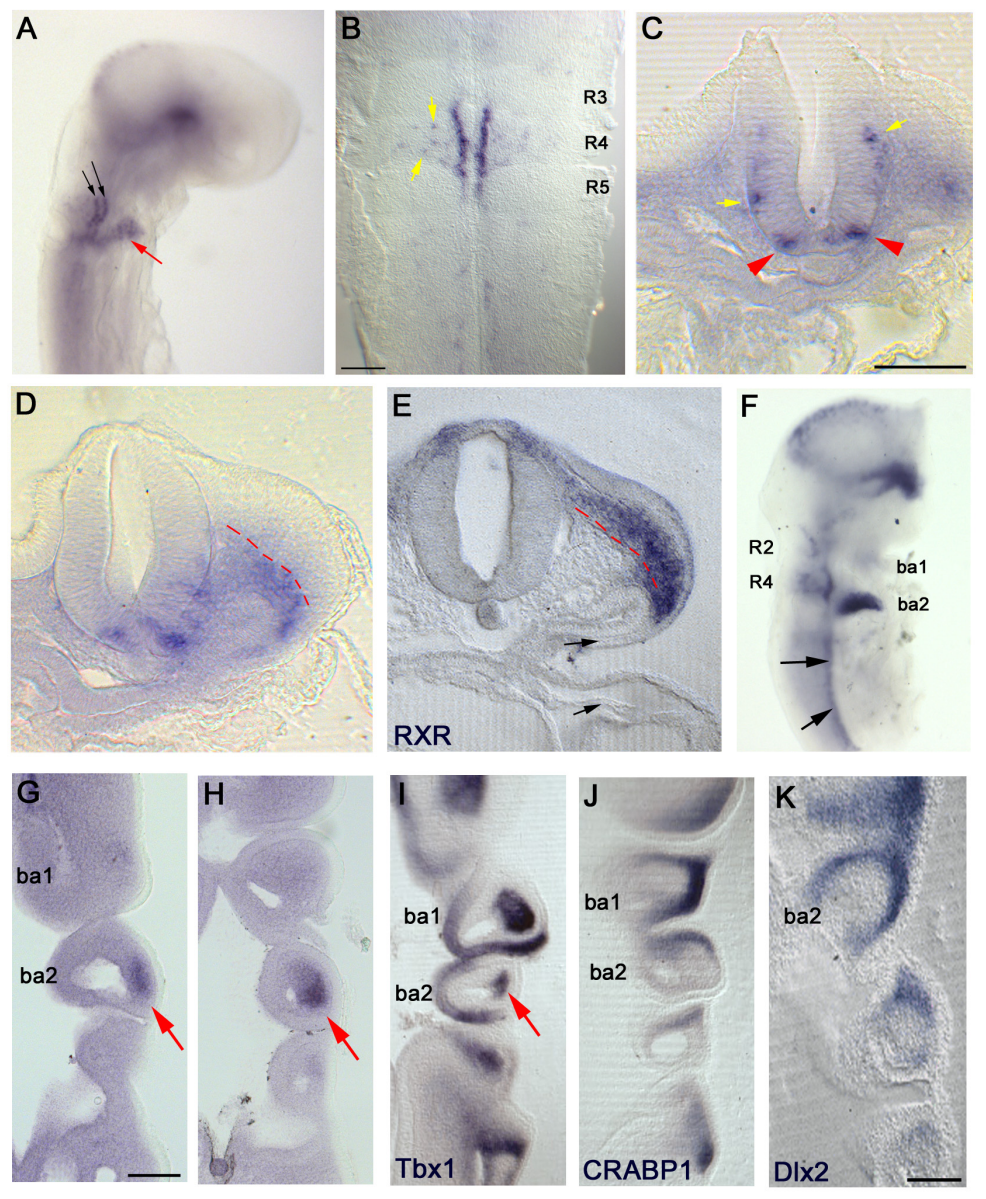
***Lrrn2 *marks rhombomere 4 motor neurons and the second branchial arch**. *In situ *hybridisation with **(A-D, F-H) ***Lrrn2*, **(E) ***RXR*, **(I) ***Tbx1*, **(J) ***CRABP1*, **(K) ***Dlx2 *at HH14 (A-E) and HH18 (F-K). (A) Wholemount embryo shows *Lrrn2 *expression in two stripes in ventral r4 (black arrows) and in nearby cells within the presumptive BA2 region (red arrow). (B) Flatmounted hindbrain shows *Lrrn2 *expression restricted to two columns immediately alongside the ventral midline of r4, with a few scattered cells within the body of r4 (yellow arrows). (C) Transverse section through the hindbrain at the level of r4 shows expression in the outer layer (red arrowheads). Yellow arrows indicate more dorsal cells seen also in (B). (D) Next posterior transverse section of the same series as (C) shows staining in the ventrally located mesoderm (below red dashed line). (E) Neural crest marker *RXR *labels a dorsally migrating population lying above the same line. (F) At HH18, *Lrrn2 *is strongly expressed in BA2 but is essentially absent from BA1. Staining is also visible in scattered cells more dorsally in r2 and r4, as well as in the ventral motor column (black arrows) and ventral midbrain. (G, H) Adjacent coronal sections through the branchial arch region at HH18 shows *Lrrn2 *in the central core area of BA2 (red arrows). (I) *Tbx1 *expression labels the mesodermal core of the branchial arches; staining in the BA2 core area is indicated by a red arrow. (J) *CRABP1 *and (K) *Dlx2 *are neural crest markers and show a peripheral crescent of staining in the branchial arches, unlike *Lrrn2*. Scale bars: 100 μm (E, I-K). Courtesy of Robyn Quinlan and Anthony Graham.

R4 is the source of a population of motor neurons that innervate BA2 muscles [37]. Analysis of *Lrrn2 *expression at HH14 (Figure 2A) shows strong staining in the CNS in a ventral domain within r4 and in non-neuronal cells in BA2. The latter could represent either r4-derived neural crest cells, which migrate into the arch at this stage, or mesodermal cells that give rise to branchial muscle. Staining is also seen in the ventral midbrain. Flatmounting of the hindbrain shows that *Lrrn2 *expression is largely restricted to cells immediately adjacent to the floor plate in r4 corresponding to the progenitor domain for r4 motor neurons. A few scattered *Lrrn2*^+ ^cells are also seen more dorsally (Figure 2B).

Motor neurons are generated at HH14, in response to Shh signalling, from progenitors located adjacent to the floor plate in the CNS ventricular zone. Differentiating, post-mitotic motor neurons move radially into the outer, mantle zone and express the transcription factor Islet-1 (Isl1) [38]. To determine whether *Lrrn2 *expression corresponded to either of these populations, transverse sections at the level of r4 were examined. *Lrrn2*^+ ^cells are located away from the ventricular surface in the mantle zone and, therefore, are likely to be post-mitotic motor neurons (Figure 2C).

Two types of motor neuron are generated in r4: the facial branchiomotor (FBM) neurons, which will innervate voluntary muscles of the second branchial arch; and the vestibular-acoustic (VA) efferent neurons, which will innervate hair cells of the inner ear. In the chick, FBM neurons migrate dorsally and extend axons ipsilaterally in a dorsolateral direction to exit the neural tube via the r4 exit point in the alar plate [37,39]. A subpopulation of VA neurons undergoes a characteristic migration in which their cell bodies migrate across the midline after they have already extended axons dorsolaterally to join FBM axons, resulting in a contralateral projection [40]. *Lrrn2*^+ ^cells seen more dorsally in r4 were also located in the mantle zone (Figure 2C). Their position is consistent with them being tangentially migrating FBM neurons that have begun their dorsal-ward migration, although this has not been demonstrated. In support of this, no *Lrrn2*^+ ^cells that could represent a contralateral VA population were seen at the midline.

*Lrrn2 *expressing cells were also observed in the periphery (Figure 2A, C, D). Cells migrating into the arches from the paraxial mesoderm are known to occupy a ventral territory while neural crest follows a more dorsal route [41]. *Lrrn2*^+ ^staining is seen in a more ventral location (Figure 2D), indicating that they are likely to represent migrating mesodermal cells rather than migratory neural crest cells, which normally occupy a dorsal position as shown by the neural crest marker *RXR *[42] at HH14 (Figure 2E).

At HH18 (Figure 2F), *Lrrn2 *expression remains intense in ventral r4 and also in more dorsolateral cells (putative FBM cell bodies). Weaker staining is also seen in r2, including a dorsal population perhaps representing the dorsally migrating trigeminal motor neurons, as well as throughout the ventral motor column in the spinal cord and in the ventral midbrain (Figure 2F). Strong staining is seen in BA2, with little or no staining in BA1 (Figure 2F). Coronal sections through the branchial arches at this stage show *Lrrn2 *in the core region of BA2 (Figure 2G, H). For comparison, corresponding sections at the same stage of mesodermal marker *Tbx1 *[43,44] and neural crest markers *CRABP1 *[45] and *Dlx2 *[46,47] are also shown (Figure 2I–K). These demonstrate that mesodermal cells are found in the central core region of BA2 (Figure 2I) while neural crest cells occupy a peripheral territory (Figure 2J, K). This indicates that at HH18 *Lrrn2 *labels mesodermal cells in BA2.

### *Lrrn2 *expression is responsive to Shh signalling only in r4

Shh is an important determinant of motor neurons at all AP axial levels of the neural tube and it is well established that artificially increasing the level of Shh signalling results in an increased generation of motor neurons [12,13]. To test whether *Lrrn2 *is induced by Shh in motor neurons, we used *in ovo *electroporation to misexpress *Shh *unilaterally in the hindbrain. A plasmid encoding chick Shh (pXeX-*Shh*) [48] was co-electroporated at HH9–11 with a green fluorescent protein (GFP) expression vector (pCAβ-eGFPm5) [49] to mark electroporated cells. Co-expression of the two plasmids was confirmed by *in situ *hybridisation with a chick *Shh *probe and antibody staining for GFP (data not shown). Embryos were analysed 24 h following electroporation.

**Figure 3:**
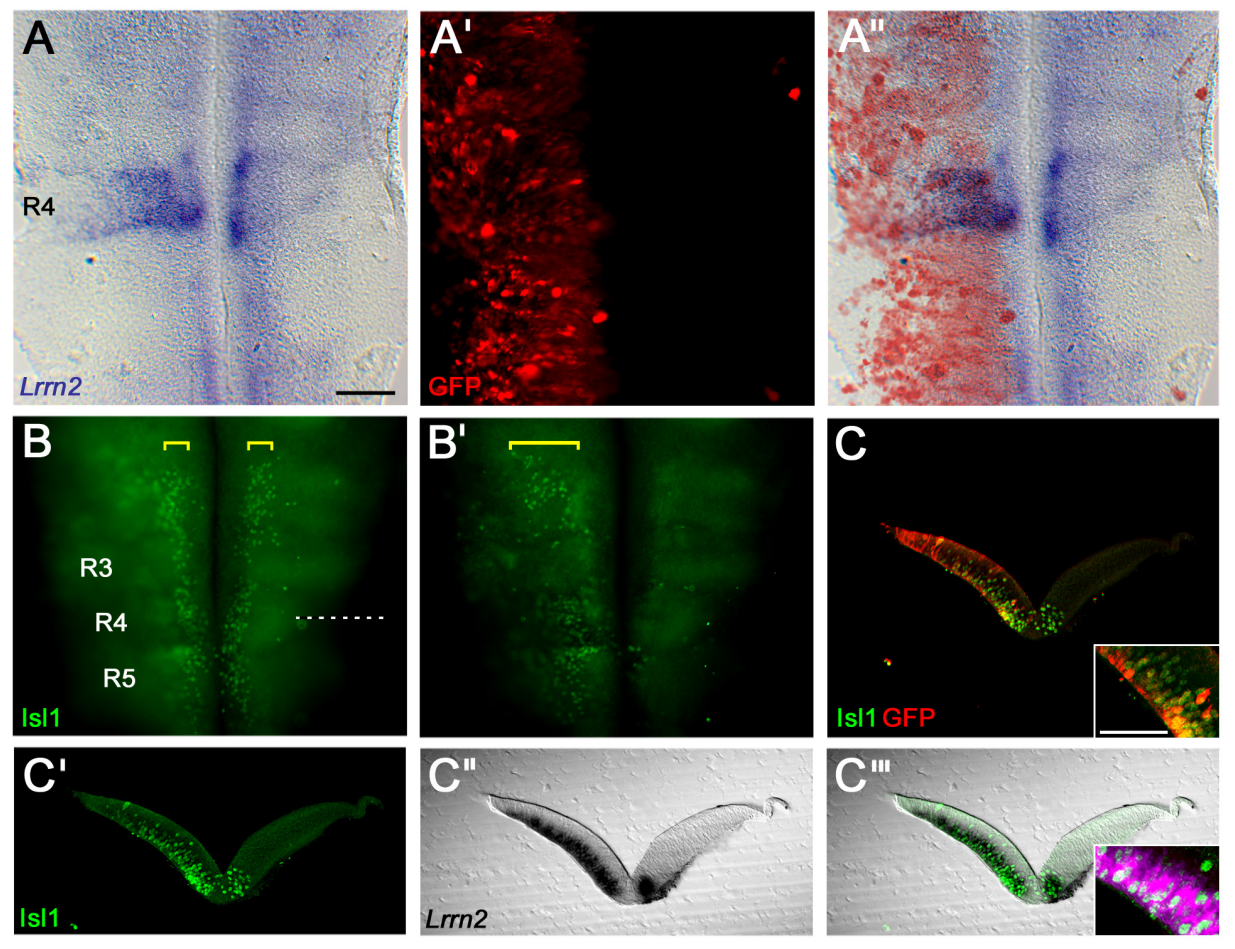
**Shh upregulates *Lrrn2 *in rhombomere 4**. **(A-A") **Co-electroporation of Shh and GFP results in overexpression throughout the left side of the hindbrain as visualised by anti-GFP labelling, reflecting a similar pattern of *Shh *misexpression (data not shown). This results in an upregulation of *Lrrn2 *expression only in r4. (A) *Lrrn2 *expression, (A') GFP expression, (A") overlay. **(B, B') **The same embryo, following immunocytochemistry with anti-Isl1/2 antibody (green) to label motor neurons, showing the normal pattern of Isl1/2 antibody staining in motor neurons lying adjacent to the floor plate (yellow brackets); (B) photo taken with focal plane set ventrally, and (B') a more dorsal focal plane showing upregulation of Isl1/2 throughout the left side of the hindbrain, as one would predict (expanded yellow bracket). Dashed line indicates the plane of section of (C-C"'). **(C-C"') **Transverse sections through the hindbrain at the level of r4. (C) Overlay of Isl1/2 in green and GFP in red, indicating unilateral Shh overexpression and induction of Isl1/2 expression. Inset shows high power image. (C'-C"') The expansion of *Lrrn2 *expression can be seen to be confined to the mantle layer (C") and colocalises with upregulation of Islet1/2 (Isl1/2 alone (C'); overlay with *Lrrn2 *(C"'); inset shows high power view with *Lrrn2 *expression pseudocoloured in magenta to more easily visualise overlap with Isl1/2 staining). Scale bar: 100 μm; 50 μm (inset).

*In situ *hybridisation for *Lrrn2 *following overexpression of *Shh *throughout the left side of the hindbrain revealed upregulation of *Lrrn2 *only within r4 (Figure 3A–C; n = 6). Thus, although at later stages *Lrrn2 *is more widely expressed at low levels in a ventral domain throughout the AP axis of the neural tube, it is only strongly responsive to Shh signalling in r4.

To confirm that the region of *Lrrn2 *upregulation in r4 corresponded to motor neurons, we used an Isl1 antibody, which labels all post-mitotic motor neurons [50]. As expected, we detected a dramatic expansion of the number of Isl1^+ ^motor neurons throughout the hindbrain. A dorsal expansion of Isl1^+ ^cells was seen on the electroporated side of the neural tube, but not on the control (unelectroporated) side (Figure 3B, B'). Sections through the hindbrain at the level of r4 demonstrate that the area of *Lrrn2 *upregulation co-labels with the Isl1 antibody (Figure 3C). Thus, while overexpression of Shh can lead to the induction of ectopic motor neurons all along the AP axis of the hindbrain, induction of *Lrrn2 *is restricted to those induced in r4. Moreover, it indicates that *Lrrn2 *is a specific marker of r4 motor neurons in the chick.

### Shh and Hoxb1 co-operate to regulate the expression of *Lrrn2*

Since *Hoxb1 *is known to confer r4 identity, we next asked whether *Lrrn2 *could be a downstream target of Hoxb1.

**Figure 4:**
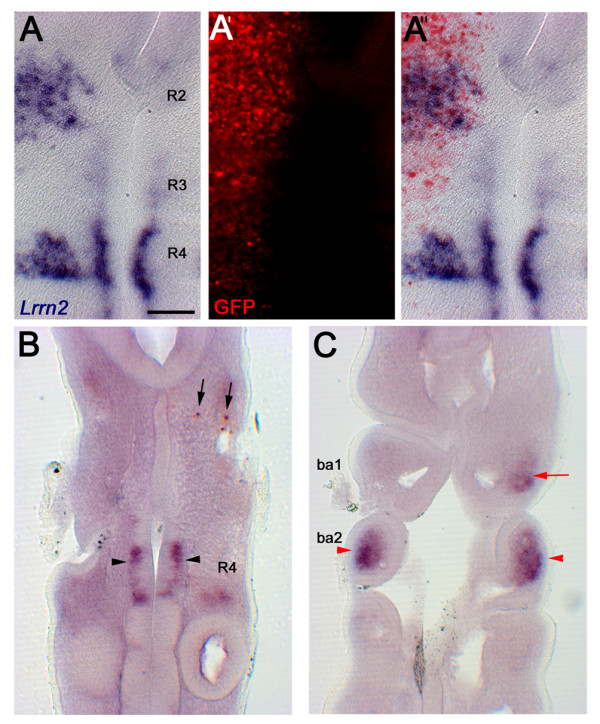
**Hoxb1 regulates *Lrrn2 *expression**. **(A-A") **Overlay to show co-expression of ectopic Shh and Hoxb1 and their effect on *Lrrn2 *expression in a flatmounted hindbrain, using an anti-GFP antibody to localise the region of misexpression. Widespread overexpression is seen throughout the hindbrain, but *Lrrn2 *is upregulated in r1, r2 and r4 only. (A) Brightfield view of *Lrrn2 *expression and (A') fluorescence of anti-GFP antibody. **(B-C) **Coronal sections through an embryo electroporated with RCASBP(B)-*mHoxb1 *at HH10 and processed 2 days later for *Lrrn2 *(dark purple) and mouse *Hoxb1 *(red) expression by *in situ *hybridisation. (B) Section taken through ventral r4 show endogenous expression of *Lrrn2 *in r4 (black arrowheads) and ectopic expression of mouse *Hoxb1 *(black arrows). (C) Corresponding section taken more ventrally through the branchial arches of the same embryo shows upregulation of *Lrrn2 *expression in BA1 mesoderm (red arrow) and endogenous expression in BA2 (red arrowheads). Scale bar: 100 μm.

An expression vector containing the mouse *Hoxb1 *coding region linked by an internal ribosomal entry site (IRES) sequence to *GFP *(pCAβ-*Hoxb1-IRES-eGFPm5*; see Materials and methods) was electroporated into the developing hindbrain at HH9 and analysed 24 h later. *In situ *hybridisation with a mouse *Hoxb1 *probe and immunohistochemistry with an anti-GFP antibody confirmed co-expression. Ectopic *Hoxb1 *expression had no effect on *Lrrn2 *but expression in embryos that survived the electroporation procedure was always dorsal to the most ventral, Shh-expressing region of the hindbrain (data not shown). We reasoned that expression of *Lrrn2 *might be dependent on Shh and, therefore, attempted to target *Hoxb1 *misexpression to the domain of endogenous Shh expression in the ventral hindbrain by modifying our electroporation technique to place the positive electrode underneath the neural tube. However, this region coincides with the location of the developing heart, and at the necessary voltages, electroporation at these stages resulted in terminal cardiac damage. We therefore co-electroporated pCAβ-*Hoxb1-IRES-eGFPm5 *with pXeX-*Shh *into more dorsal regions of the hindbrain. The combined expression of Shh and Hoxb1 in the hindbrain resulted in the upregulation of *Lrrn2*, but only in r1, r2, and r4 (Figure 4A–A"; n = 4). Despite the combined expression of both plasmids in r3 and r5, no induction of *Lrrn2 *was seen in these two rhombomeres. Therefore, *Lrrn2 *is a likely target of Hoxb1 and the co-expression of Hoxb1 with Shh is sufficient for its induction in r1, r2 and r4 neuroepithelium.

### Hoxb1 can regulate the mesodermal component of *Lrrn2 *expression

*Hoxb1 *is normally expressed in both the r4 neuroepithelium and r4 neural crest, but not in the mesoderm, so any effect on the mesodermal expression of *Lrrn2 *would be indirect. However, there is strong evidence to suggest the existence of interactive signalling between all three tissues during branchial arch patterning [51,52]. To see if the mesodermal expression of *Lrrn2 *in BA2 was responsive to the non-cell autonomous actions of Hoxb1, we overexpressed *Hoxb1 *in the hindbrain at HH10 and analysed embryos 48 h later. We saw no effect on the BA component of *Lrrn2 *expression when using pCAβ-*Hoxb1-IRES-eGFPm5 *for over-expression (not shown). However, this could have been a result of the significant dilution due to tissue growth that occurs over this period.

To obviate this possibility, we electroporated mouse *Hoxb1 *in the hindbrain region at HH9–11 using a plasmid encoding the retroviral vector RCASBP(B)-*Hoxb1 *[6]. Embryos were analysed 2 days later for the expression of mouse *Hoxb1 *and *Lrrn2*, and sectioned on the coronal plane to visualise the cell populations of the branchial arches. *Hoxb1 *misexpression resulted in the induction of *Lrrn2 *in BA1 (Figure 4B, C; n = 4). Similarly to the endogenous situation in BA2, ectopic BA1 *Lrrn2 *expression was confined to the inner core of branchial arch mesenchyme (Figure 4C; compare with BA1 staining of mesodermal marker *Tbx1 *in Figure 2I). These results indicate that Hoxb1 is able to ectopically upregulate the mesodermal expression of *Lrrn2 *in BA1.

### Mistargeting of r2/3 neurons expressing ectopic Lrrn2 to the second branchial arch

The predicted secondary structure of Lrrn2 indicates that it is a single-pass transmembrane protein and a component of the plasma membrane, as we have previously shown for the closely related Lrrn1 protein [32]. This is supported by biochemical and immunocytochemical data confirming it as a glycosylated protein and a component of the endoplasmic reticulum and Golgi complex [33]. A number of related proteins containing extracellular leucine-rich repeats have been shown to play an important role in cell adhesion and axon guidance [28-30,53-55]. A previous study has demonstrated that ectopic expression of *Hoxb1 *in basal r2 can result in the misprojection of r2 motor neurons [6] and we have now shown that *Lrrn2 *is downstream of Hoxb1. Since *Lrrn2 *is specifically expressed at a high level in r4 motor neurons and in their target tissue, the mesodermal pre-muscle mesenchyme of BA2, we postulated that it might function downstream of Hoxb1 in the guidance of r4 motor axons to their target pre-muscle region.

**Figure 5:**
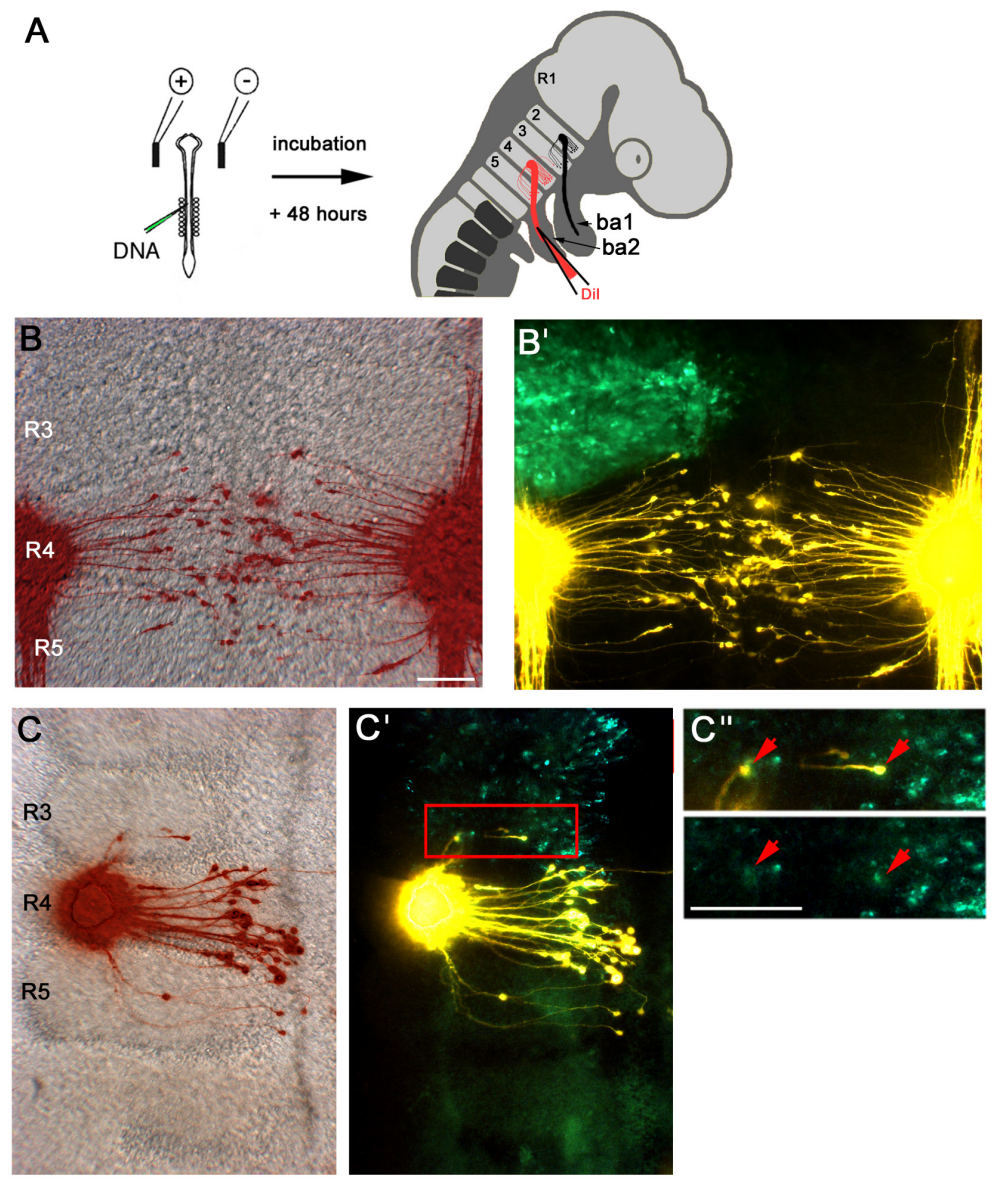
**Ectopic Lrrn2 can cause misrouting of axons**. **(A) **Schematic to show experimental approach. On the left is shown a HH10 chick embryo with electrodes positioned on either side of the hindbrain. DNA is injected into the neural tube and following electroporation is expressed unilaterally on the side of the positive electrode. After 48 h embryos are harvested and the retrograde dye DiI is injected into BA2. Dye travels along axonal processes and labels the cell bodies from which they originate. **(B, B') **Flatmounted hindbrain from a control embryo electroporated with pCAβ-*eGFPm5 *shows motor projections to BA2 labelled by retrograde DiI injection. (B) Overlay of artificially coloured DiI fluorescence (red) showing BA2 axons and a brightfield photograph of the hindbrain. (B') Overlay showing strong GFP expression in r2 and r3 on the left side of the hindbrain. All axons projecting to BA2 clearly originate in r4 and r5 with no cell bodies lying anterior to the r3/4 boundary. **(C, C') **Embryo with misexpression of Lrrn2-GFP in r2/3 shows several ectopic axons that project from cell bodies in r3 to BA2. **(C") **High power views of the boxed region in (C') showing that the ectopic axons originate from Lrrn2-GFP^+ ^cells (red arrows). Scale bars: 100 μm.

To test this hypothesis, we misexpressed *Lrrn2 *in r2/3 by electroporation and analysed the routing of axons to BA2 via retrograde tracing. A vector expressing the full-length Lrrn2 protein, fused at its intracellular carboxyl terminus to GFP (CAB-*Lrrn2-eGFPm5*; see Materials and methods), was electroporated unilaterally into the hindbrain at HH10–15. Embryos were harvested 2 days later, by which time motor projections have reached their targets in the branchial arches. Motor neuron axonal projections to BA2 were visualised by injecting the retrograde tracer DiI into BA2, so that all cell bodies with projections to BA2 are clearly labelled (Figure 5A).

Control electroporations using a GFP expression construct (pCAβ-*eGFPm5*) to misexpress GFP only in r2/3, did not result in any mis-targeted axons (Figure 5B, B'; n = 15). However, misexpression of Lrrn2-GFP in r2/3 over the same period resulted in a number of mistargeting events in embryos that showed intermediate-to-high levels of Lrrn2-GFP expression (Figure 5C–C"; n = 4/5; total 14 cells misrouted to BA2, 4 of 14 from r2, 10 of 14 from r3). This was, however, a rare event: of the four embryos with rerouted axons, only 2.5% (14 of 569) of Lrrn2-GFP^+ ^cells in r2/3 showed mistargeting to BA2. Several embryos (five of five) with low-level expression (only a few scattered Lrrn2-GFP^+ ^cells were visible) did not exhibit a rerouting phenotype. This phenotype may have been due to a community effect [56,57]. To investigate this further, we carefully examined the localization of Lrrn2-GFP-expressing cells in relation to misrouting. In total, approximately 30% of misrouted neurons (4 of 14) were surrounded by other Lrrn2-GFP^+ ^cells and of the remaining 70% (10 of 14), all showed at least one Lrrn2-GFP^+ ^cell adjacent to the cell body of the misrouted neuron. These experiments indicate that ectopic Lrrn2 expression in r2/3 is sufficient to cause ectopic targeting of r2/3 axons to BA2 and, therefore, suggests that its normal role may be to participate in guiding facial motor axons to their target pre-muscle cells in BA2.

## Discussion

In summary, *Lrrn2 *is expressed at a high level in post-mitotic r4 motor neurons, and their target cells, the mesoderm-derived pre-muscle core of BA2. Hoxb1 and Shh co-operate to regulate *Lrrn2 *expression in r4 motor neurons, while Hoxb1 alone can upregulate *Lrrn2 *ectopically in the mesodermal core of BA1 via a non-cell autonomous mechanism. Furthermore, misexpression of Lrrn2 in r2/3 can lead to axonal routing abnormalities, particularly ectopic projections from r2/3 neurons to BA2.

### Hoxb1 and Shh regulate the expression of *Lrrn2*

*Hoxb1 *acts as a selector gene to control r4 identity [6,7,9] and thereby regulates, either directly or indirectly, a network of genes that all contribute to different aspects of r4 identity, including downstream transcriptional regulatory cascades and effectors of cellular behaviour. We have demonstrated that *Lrrn2 *is positively regulated by *Hoxb1 *and suggest that it represents one of these elusive effector genes. We have not yet addressed whether *Hoxb1 *and *Shh *act directly or indirectly on *Lrrn2 *expression and the molecular details underlying these interactions remain unknown. The *Hoxb1 *r4 autoregulatory enhancer has been well characterised in mouse and chick and consists of a number of conserved binding sites for Hoxb1, Pbx1 and Prep1 [58-60]. We have conducted a preliminary search of the ± 10-kb region flanking the chick *Lrrn2 *gene but have not identified any similar sequences that could represent *Lrrn2 *r4 regulatory elements. However, the chick genome sequence is incomplete and it remains possible that *Hoxb1 *could regulate *Lrrn2 *directly. Alternatively, given the regulation of expression in BA1 mesoderm is non-cell autonomous, it may act indirectly to regulate *Lrrn2 *in r4. There is only limited information available on downstream target genes of *Hoxb1*. Although *Hoxb1 *is known to directly regulate both itself [59], *Hoxb2 *[61] and *Hoxa2 *[62], previous work using electroporation to misexpress Hoxb1 ectopically in the chick hindbrain showed no upregulation of these genes [63], making these unlikely to act as intermediaries. *GATA2 *and *GATA3 *are transcription factors known to be expressed in r4 and regulated by *Hoxb1*, although it is not known if they represent direct targets [6,17,18]. Interestingly, uniform overexpression of *Hoxb1 *results in the upregulation of *GATA2 *only in r2 [18], which would be consistent with it acting as an intermediary to regulate *Lrrn2*.

Ectopic expression of Hoxb1 in the motor neuron progenitor domain of r1 is known to induce Isl1 expression in this region, which is usually devoid of Isl1 staining [63]. This has been presumed to be due to a homeotic transformation of r1 into an r4-like character [63]. However, it is not known if this is a direct effect and, as we have shown for *Lrrn2*, Isl1 induction probably requires Shh signalling since no Isl1 expression was seen outside the domain of endogenous Shh influence [63]. An example of a direct target of *Hoxb1 *is the paired-like homeodomain transcription factor *Phox2b*, which is expressed in FBM neurons [19]. However, as both Isl1 and Phox2b are strongly expressed throughout the AP axis of the hindbrain and do not show r4-specific expression, they cannot mediate r4 specificity of *Lrrn2 *expression.

Although the regulation of *Lrrn2 *by *Hoxb1 *may be indirect, it represents a strong candidate for a downstream effector gene, of which very few have been described. A recent microarray screen in zebrafish searching for downstream targets of *zfHoxb1a *has identified 12 genes, including *prickle1b*, which is required for FBM neuron migration [20]. It is not yet known whether any or all of these zebrafish *Hoxb1a *targets are direct or indirect [20] but, like *Lrrn2*, they critically display r4 specificity and are, therefore, likely to be involved in generating r4-specific identity and cell behaviours.

Similarly, regulation of *Lrrn2 *by Shh could be at the level of its transcriptional effectors, the Gli family members [64-66], or further downstream. For example, Nkx2.2 is expressed in the appropriate branchiomotor progenitor domain and, although it is rapidly lost from post-mitotic neurons, could be involved in the induction of *Lrrn2 *expression [11,15]. The expression domain of *Lrrn2 *in r4 corresponds closely to those of Isl1/2 (Figure 3) [67] and Phox2b [68]. These are, therefore, candidate intermediaries for the action of Shh on *Lrrn2*. Both Isl1/2 and Phox2b are critical for the development of FBM neurons. Isl1 is an early marker for motor neurons [50] and is required for motor neuron generation [69]. Loss of *Phox2b *leads to a failure in axon extension and migration of FBM neurons followed by extensive cell death [70,71].

The ectopic expression of Hoxb1 alone was able to upregulate the expression of *Lrrn2 *in BA1 mesoderm. This suggests that the endogenous BA2 expression of *Lrrn2 *may be Hoxb1-dependent. Since Hoxb1 is not normally expressed in the branchial arches, and only in the neurogenic r4-derived neural crest [2,72], any normal regulation of the mesodermal expression of *Lrrn2 *must be indirect and involve an unknown secreted signal, with a mechanism that may be distinct from that operating in r4 motor neurons. This is consistent with the absence of ectopic *Hoxb1 *in the cells expressing ectopic *Lrrn2*. It has been shown that the cranial neural crest, mesoderm and their surrounding epithelial tissues signal important patterning information to each other, suggesting close interdependence in arch patterning [51,52,73-77]. Furthermore, experiments using chimaeric embryos containing a mixture of wild-type and *Hoxb1*^-/- ^cells have demonstrated that Hoxb1 is required in the neural crest prior to delamination to establish and maintain the facial motor neuron circuit [78]. We hypothesise that ectopic Hoxb1 expressed in r2 neural crest may induce homeotic changes in BA1, including induction of BA2 mesodermal markers like *Lrrn2*.

### Lack of up-regulation of *Lrrn2 *by Hoxb1 and Shh in r3 or r5

The combination of Hoxb1 and Shh was not able to induce *Lrrn2 *expression in r3 and r5. It has been shown that in transgenic mice that express Hoxb1 ubiquitously, *GATA2 *is ectopically upregulated only in r2 [18], while previous data from our laboratory have shown that Hoxb1 misexpression throughout the hindbrain can induce strong, r4-like expression of the immunoglobulin superfamily cell surface glycoprotein BEN in r2 but not in r3 or r5 [6]. Moreover, while in some embryos r2 motor axons projected inappropriately to BA2, almost all Hoxb1-expressing r3 motor neurons retained their correct projection to BA1 [6]. It seems, therefore, that r3 motor neurons are refractory to the effects of ectopic Hoxb1 expression. Our results provide the possible explanation that this could be due to a lack of induction of *Lrrn2*, which would act as a guidance factor for BA2-targeted axons. It is possible that r3 and r5 can exert repressive effects downstream of Hoxb1 by acting on its target genes. A potential candidate for such an effect is the zinc finger transcription factor Krox20, which is expressed in r3 and r5 from early stages. It is clear that there are fundamental differences between odd and even numbered rhombomeres. The identification of *Lrrn2 *as a downstream target of *Hoxb1 *makes it a useful tool to further dissect the regulation of this odd-even periodicity.

### Ectopic Lrrn2 in r2/3 can lead to rerouting of axons to BA2

While misexpression of Lrrn2 in r2/3 resulted in the mis-projection of some axons to the r4 exit point and BA2, this was a rare event and we did not observe the dramatic rerouting seen by Bell *et al*. [6] when misexpressing Hoxb1 in r2. This may be due to a number of factors associated with the different methodologies used. Electroporation frequently leads to lower levels and a mosaic distribution of expression compared to viral misexpression. In support of this idea, other investigators have commented on the necessity for 'very efficient electroporation' [79], where the operation of dominant community effects may reinforce the status quo [57,80]. Alternatively, the developmental stage at which the experiments were carried out may have affected the outcome. Retroviral Hoxb1-misexpression by Bell *et al*. was carried out at HH3 while our experiments were performed at HH10–14. It is possible that later in development cells have become committed to a specific fate that cannot be altered. For example, r5 somatic motor neurons become committed to their fate between HH9 and HH10 [81]. Consistent with this, electroporation of Hoxb1-IRES-GFP at HH10–14 into r2/3 did not lead to any routing abnormalities (n = 16, data not shown). Unfortunately, attempts to use either higher voltages or earlier stages were not compatible with survival. It is also likely that other guidance molecules are involved, particularly at interim decision points in outgrowth and pathfinding, such as to find the correct exit point from the hindbrain [82]. The absence of these in Lrrn2 misexpressing cells may have contributed to the low incidence of rerouting.

### Lrrn2 and neuromuscular targeting: a conserved role from *Drosophila*?

One of the most striking aspects of *Lrrn2 *expression is the correlation between a particular subset of hindbrain motor neurons, the facial branchiomotor neurons of r4, and their prospective muscle targets, the core mesoderm cells of BA2. Although cell adhesion/recognition molecules that display such a correspondence have been identified in invertebrates, very few have been found in vertebrates. In a tantalising parallel, one of the closest *Drosophila *homologues to the Lrrn proteins is Caps, which is expressed on a subset of motor neurons and their muscle targets, and is known to play a role in regulating target specificity of motor neurons [30,31]. In *caps *mutants, axon targeting for one of these matching pairs is abnormal. Pan-muscle expression of Caps results in the muscle 12 motor neurons forming ectopic synapses on a nearby muscle, while pan-neural expression of Caps leads to pathfinding errors in the same motor neurons [30,83]. This may in part be mediated by a sensing function of Caps expressed on muscle filopodia to enable synaptic matching [31]. Caps has also been shown to mediate specific axon-target interactions in the *Drosophila *visual system, where it is expressed in R8 photoreceptors and their target layer, and regulates this layer-specific targeting [55]. Another closely related LRR molecule, Connectin, plays a similar role in another set of motor neurons and their muscle targets [28,29,54], and has been identified as one of the few known cell adhesion molecules to be directly regulated by a member of the *Hox*/HOM-C complex, Ubx [84,85].

Cues acting on generic features of motor neuron development have been identified [21-23,86]. However, knowledge about specific factors that endow individual identity to a motor neuron and specificity for its target muscle group is lacking. It is tempting to speculate that Lrrn2 might function in an analogous manner to *Drosophila *Caps in the specific targeting of facial branchiomotor neurons to the muscles of the BA2. To determine if this is the case, an experimental design would be required that would drive Lrrn2 expression in r2 motor neurons from an early developmental stage to look for rerouting, and, in a complementary approach, expression would be driven in BA1 mesoderm to see if r4 FBMs ectopically synapsed on to BA1 muscles. However, this is not yet feasible in the chick with current techniques.

*Lrrn2 *is also strongly expressed in a number of other sites in the developing chick embryo, such as the region on the right-hand side of Hensen's node and the ventral midbrain, where its role remains unknown. If *Lrrn2 *has a role specific to r4, could other related proteins serve similar functions in different regions of the neuraxis, perhaps downstream of other *Hox *genes? *Lrrn2 *is part of a three-member gene family in chick and mouse [32,33], but neither *Lrrn1 *[32] nor *Lrrn3 *(unpublished data) show expression patterns in the chick obviously consistent with this. However, there is a very large extended family of closely related genes (in the fibronectin leucine-rich transmembrane (FLRT) family, leucine-rich transmembrane family (LRRTM/LRTM) and LINGO/LRRN6/LERN family [87-90]), members of which might well perform similar roles in other cell types and species.

## Conclusion

We have identified a novel cell surface receptor in the chick, *Lrrn2*, which is specifically expressed by r4 motor neurons and their prospective target tissue, the mesodermal pre-muscle core of BA2. Its expression in r4 motor neurons is regulated by Hoxb1, in co-operation with the signalling molecule Shh. Misexpression of Lrrn2 in r2/3 results in ectopic axonal projections to the r4 exit point and BA2, indicating that Lrrn2 may be a candidate for regulating specific motor axonal targeting in the vertebrate hindbrain.

## Materials and methods

### Chick methods

Chicken eggs (Rhode Island Red) were incubated at 38°C in 40 to 50% humidity until the appropriate stage was reached. Embryos were staged according to Hamburger and Hamilton [91].

### *In ovo *electroporation

Chick eggs were incubated until the appropriate stage was reached. Electrodes were made from silver wire (diameter 0.5 mm), platinum-iridium wire (80:20 mix, diameter 0.25 or 0.5 mm) or tungsten wire (diameter 0.125 mm) and placed at the desired position relative to the embryo. DNA at 0.5 to 1.0 μg/μl in water containing 0.1% Fast Green to aid visualization was pressure injected into the lumen of the neural tube using a glass capillary micropipette. Square wave pulses of 8 to 20 V (1 to 6 pulses of 50 ms) were delivered to the tissue using an Intracept TSS10 Dual Pulse isolated stimulator (Intracel, Royston, Herts, UK). The electrodes were removed and a few drops of Tyrode's solution (containing 137 mM NaCl, 2.7 mM KCl, 0.32 mM NaH_2_PO_4_.2H_2_O, 2.4 mM CaCl_2_, 1.0 mM MgCl_2_, 5.5 mM glucose) were added. Eggs were resealed and returned to the incubator for 24 to 48 h before harvesting.

To generate the Hoxb1-IRES-GFP expression vector, the coding sequence of mouse *Hoxb1 *was excised from RCAS(BP)B-*Hoxb1 *[6] as a *Cla*I fragment and cloned into the vector CA-IRES *eGFPm5*. This consists of the hybrid cytomegalovirus enhancer-chick β *actin *promoter vector pCAGGS [92,93] containing the encephalocardiomyopathy virus (ECMV) IRES and a modified polylinker (A Hunter and J Gilthorpe, details available upon request). The correct insert orientation was verified by restriction endonuclease digestion. The CAB-*Lrrn2-eGFPm5 *fusion vector was generated by PCR amplification of the Lrrn2 coding region, without the stop codon, using the plasmid template used to generate the *Lrrn2 in situ *probe (see below) and cloning into the pCR4-TOPO vector (Invitrogen, Carlsbad, CA, US). Following DNA sequencing to verify the sequence of the insert, it was cloned into pCA-*eGFPm5 *(details available upon request). Other expression vectors used were: pCAβ-*eGFPm5 *[49], pXEX-*cShh *(gift of C Ragsdale) [48], RCASBP(B)-*mHoxb1 *[6].

### Retrograde labelling of axons with DiI

Embryos were fixed with 4% paraformaldehyde (PFA) and pinned down in a Sylgard-coated dish (Dow Corning, Midland, MI, US). DiI (1,1'-didodecyl-3,3,3',3'-tetramethylindocarbocyanine perchlorate; Invitrogen) was pressure injected via a glass capillary micropipette into the dorsal-proximal region of the second branchial arch. Embryos were kept in the dark at room temperature for 1 week in PFA and then examined under a fluorescence dissecting microscope before flatmounting hindbrains.

### *In situ *hybridisation

Chick embryos were fixed overnight at 4°C in PFA. Single and double *in situ *hybridisations were performed as described previously [32] except that hybridisation with the *Lrrn2 *probe was carried out at 72°C. Chick *Lrrn2 *(gift of F Murray, Roslin Institute, Roslin, Edinburgh) [GenBank:AL588402] was linearized with *Sal*I and transcribed with SP6 RNA polymerase (Roche Applied Science, Burgess Hill, UK) to generate an antisense probe. Additional plasmid templates were used to generate antisense riboprobes as follows: chick *Shh *(*Sal*I-SP6; gift of T Lints); chick *Hoxb1 *(*Xba*I-T7; gift of V Prince), mouse *Hoxb1 *(*Hind*III-T7; gift of R Krumlauf).

### Wholemount immunohistochemistry

Following *in situ *hybridization, embryos were washed and blocked in phosphate-buffered saline (PBS) containing 1% new born calf serum for 3 × 45 minutes at room temperature. Embryos were incubated in primary antibody (diluted in PBS/1% new born calf serum containing 0.1% tritonX-100 (PBST) for 2 to 3 days at 4°C and then washed three times in PBST for 60 minutes each at 4°C. They were then transferred into fluorescently conjugated secondary antibody (Alexa Fluor^®^, Invitrogen; goat anti-rabbit or anti-mouse depending on primary), diluted 1:200 in PBS/1% newborn goat serum/0.1% tritonX-100, and incubated overnight at 4°C. Embryos were washed three times for 45 minutes each at room temperature. Primary antibodies used were rabbit anti-GFP (Invitrogen) at 1:1,000 and mouse anti-Islet-1 (4D5, Developmental Studies Hybridoma Bank) at 1:10.

### Flatmounting and photography

Embryos were cleared in 80% (v/v) glycerol prior to digital photography of wholemounts (Olympus DP70 CCD camera). Some specimens were dissected and flatmounted in glycerol under a No. 1.5 coverslip. Vibratome (Leica VT 1000S) sections were cut at 40 μm after embedding tissue in 20% (w/v) gelatine/PBS. Following infiltration in gelatine at 65°C for 1 to 2 h, tissue was embedded and then post-fixed in PFA. Flatmounts and sections were viewed on a Zeiss Axiophot microscope and digitally photographed with a Zeiss Axiocam. Confocal images were taken on an Olympus Fluoview FV500 laser scanning confocal microscope using Fluoview software.

## Abbreviations

AP: anterior-posterior; BA: branchial arch; Caps: Capricious; CNS: central nervous system; DV: dorsal-ventral; FBM: facial branchiomotor; GFP: green fluorescent protein; HH: Hamburger and Hamilton stage; IRES: internal ribosomal entry site; Isl: Islet; Lrrn: leucine-rich repeat neuronal; PBS: phosphate-buffered saline; PFA: paraformaldehyde; r: rhombomere; Shh: Sonic hedgehog; VA: vestibular-acoustic.

## Competing interests

The authors declare that they have no competing interests.

## Authors' contributions

LCA participated in the conception and design of the study, performed the experiments and drafted the manuscript. JDG generated the CAB-*Lrrn2-eGFPm5 *construct. AL and JDG participated in the conception and design of the study and helped draft the manuscript. All authors read and approved the final manuscript.
